# DNA Hypermethylation-Regulated CX3CL1 Reducing T Cell Infiltration Indicates Poor Prognosis in Wilms Tumour

**DOI:** 10.3389/fonc.2022.882714

**Published:** 2022-04-22

**Authors:** Tao Mi, Liming Jin, Zhaoxia Zhang, Jinkui Wang, Mujie Li, Chenghao Zhanghuang, Xiaojun Tan, Zhang Wang, Xiaomao Tian, Bin Xiang, Dawei He

**Affiliations:** ^1^ Department of Urology, Children’s Hospital of Chongqing Medical University, Chongqing, China; ^2^ Chongqing Key Laboratory of Children Urogenital Development and Tissue Engineering, Chongqing, China; ^3^ China International Science and Technology Cooperation Base of Child Development and Critical; National Clinical Research Center for Child Health and Disorders, Chongqing; Ministry of Education Key Laboratory of Child Development and Disorders; Chongqing Key Laboratory of Pediatrics, Chongqing, China

**Keywords:** Wilms tumour, chemokine, immune infiltration, T cells, DNA methylation

## Abstract

**Objective:**

To investigate the role of chemokines in Wilms tumours, especially their chemotaxis to immune cells and the role of DNA methylation in regulating the expression level of chemokines.

**Methods:**

RNAseqV2 gene expression and clinical data were downloaded from the TARGET database. DNA methylation data were downloaded from the GEO and cBioPortal database. The difference analysis and Kaplan-Meier(KM) analysis of chemokines were performed by edgeR package. Then predictive model based on chemokines was constructed by lasso regression and multivariate COX regression. ROC curve, DCA curve, Calibration curve, and Nomogram were used to evaluate the prognostic model. MCPcounter and Cibersort algorithm was used to calculate the infiltration of immune cells in Wilms tumour and para-tumour samples. Then the difference analysis of the immune cells was performed. The relationship between chemokines and immune cells were calculated by Pearson correlation. In addition, DNA methylation differences between Wilms tumour and para-tumour samples was performed. The correlation between DNA methylation and mRNA expression was calculated by Pearson correlation. Western blot(WB)and immunofluorescence were used to confirm the differential expression of CX3CL1 and T cells, and the correlation between them.

**Results:**

A total of 16 chemokines were differentially expressed in tumour and para-tumour samples. A total of seven chemokines were associated with survival. CCL2 and CX3CL1 were positively correlated with prognosis, while high expression of CCL3, CCL8, CCL15, CCL18 and CXCL9 predicted poor prognosis. By lasso regression and multivariate COX regression, CCL3, CCL15, CXCL9 and CX3CL1 were finally included to construct a prediction model. The model shows good prediction ability. MCPcounter and Cibersort algorithm both showed that T cells were higher in para-tumour tissues than cancer tissues. Correlation analysis showed that CX3CL1 had a strong correlation with T cells. These were verified by Weston blot and immunofluorescence. DNA methylation analysis showed that various chemokines were different in para-tumours and tumours. CX3CL1 was hypermethylated in tumours, and the degree of methylation was negatively correlated with mRNA expression.

**Conclusion:**

1. There is low T cell infiltration in nephroblastoma. 2. Chemokines such as CX3CL1 indicate a favourable prognosis and positively correlate with the number of T cells. 3. chemokines such as CX3CL1 are negatively regulated by DNA hypermethylation.

## Introduction

Wilms tumour is a common abdominal malignant tumour in children, accounting for 90% of renal tumours in children aged 0-14 years (1). Surgery combined with radiotherapy and chemotherapy has made significant progress in treating nephroblastoma, with a 5-year overall survival rate of 90% (1, 2). However, there are still some recurrent subgroups (3). Some cases remain challenging to heal, and new treatment options need to be explored (2). At present, the treatment of cancer has entered a new era. The discovery of PD1 and PDL1 brought immunotherapy into a new field. Nivolumab and Pembrolizumab are the main PD-1 inhibitors that have been approved in clinical practice (4). They are humanized IgG4 antibodies targeting PD-1 with high affinity, Approved for non-small cell lung cancer metastatic melanoma (4). However, none of these new methods has been applied in Wilms tumours. The progress of nephroblastoma treatment seems to enter a plateau. Fresh calls have been made to explore targeted therapies and immunotherapy (2).

Immune checkpoint inhibitors have been successful in various adult tumours but are rarely reported in children with solid tumours. The causes of poor immunotherapy in children with tumours are related to ‘ cold tumours’, which are known as tumours that lack infiltration of effector CD8+ T cells or include massive accumulation of Tregs that suppress their activities (5, 6).

Despite this immune deficiency, this does not imply that immunotherapy is useless for ‘cold tumours’. There are two complementary solutions in response to this part of the patients with ineffective immunotherapy. One focuses on accurate identification of immune therapy sensitive patients. Another is committed to converting immune-insensitive patients into immune-sensitive patients or combining them with traditional treatment methods (5). For the second strategy, some successes have been achieved. For example, U3 - 1402 can enhance the infiltration of immune cells to enhance the therapeutic effect of PD1 monoclonal antibodies (7). More extensive research has focused on interfering with chemokines to induce immune cell infiltration (5, 8, 9). GPR182 ablation increases the concentration of various chemokines in tumours, making tumours with poor immunogenicity sensitive to immune checkpoint blockade and excessive cell therapy (10). Those inspired our interest to explore the role of chemokines in nephroblastoma.

Chemokines are a class of secretory inflammatory cytokines with the directional movement of chemokine cells. The primary function is to manage the migration of white blood cells to their respective positions during inflammation and homeostasis (11). According to the amino acid particular structure and conserved sequence of cysteine amino acid residues, they are divided into four categories: XC, CC, CXC and CX3C. These chemokines mainly produce a series of biological effects by binding to the homologous receptors of the G protein-coupled receptor (GPR) family located on the cell membrane surface. They are widely involved in the physiological functions of cell growth, development, cellular immunity and humoral immunity and play an essential role in various pathological processes, such as the aggregation of lymphocytes to inflammatory sites, HIV infection, and tumour growth and metastasis (11).

Chemokines play an essential role in tumour metastasis, homing and regulating the tumour microenvironment (9, 12). In different types of cancer, the role of chemokines in tumours is controversial because chemokines have the effects of both promoting and inhibiting cancer (9, 13–15). This dual role of chemokines may be associated with the chemotaxis to immune cells (8). For example, in Mouse skin melanoma cancer, CX3CL1 kills tumour cells by chemotaxis of NK cells and T cells (16). However, the role of chemokines in nephroblastoma and their chemotaxis to immune cells is not clear. Here, we analyzed the chemokines and immune cell infiltration in nephroblastoma and the potential relationship between them. In addition, we analyzed the regulation of DNA methylation on chemokine expression.

## Materials and Methods

### Data Download and Processing

The nephroblastoma (TARGET-WT) mRNA expression and clinical data were downloaded by the TCGAbiolink package of R software (R4.0.1) from the TARGET database. Screening criteria are as follows: 1. From primary tumours and para-tumour samples. 2. There are both mRNA expression data and clinical data. A total of 130 samples were obtained, including 124 tumour samples and 6 para-tumour samples. The clinical data collected included gender, age at diagnosis, stage, survival status and survival days. We downloaded the corresponding DNA methylation-related data from the cBioPortal database. DNA methylation data and mRNA expression data came from the same cohort of patients. A total of 118 samples both had DNA methylation data and mRNA expression data. In addition, another DNA methylation data were downloaded from the GEO database (GSE163372), including a total of 7 pairs of primary tumours and para-tumour.

### Analysis of Differentially Expressed Chemokines

We used raw counts for the difference analysis.The genes with an average expression level of less than 3 were removed. And finally, we obtained 32 chemokines that were expressed in Wilms tumour. These chemokines are used for differential analysis. | Log2FC | > 1 and p < 0.05 were considered significant. Peatmap and ggplot2 packages were used to draw a pheatmap.

### KM Analysis

Survival and Survminer R packages were used for KM analysis, and the samples were divided into high and low groups according to the median expression value. p < 0.05 was considered statistically significant.

### Construction and Validation of Prediction Model

To find the chemokines most related to prognosis and solve the collinearity problem, all chemokines were included in the lasso regression, and the parameters were set as family = ‘cox’, nlambda = 100. The variables screened by lasso were used for multiple stepwise regression. The R packages used here were Glmnet and Survminer. To verify the model’s prediction ability, the ROC curve, Calibration curves, and DCA curve were used. In addition, to verify whether the prediction model is independent of clinical information, we further conducted multiple stepwise regression based on the risk scores, gender, age at diagnosis and stage, then we draw a nomograms through the rms package.

### Immune Infiltration

There are mainly two algorithms to evaluate immune infiltration based on gene expression data. One is the ssGSEA-like algorithm based on marker genes, represented by MCPcounter (17). The other algorithm is based on deconvolution, and the classic one is Cibersort (18). They were both used to infer the infiltration of immune cells in Wilms tumour samples. Visualization mainly uses the ggplot2 package.

### Correlation Analysis Between Chemokines and Immune Cells

The correlation between immune cells and chemokines were analyzed by Pearson correlation. The Ggcorrplot package was used to calculate correlation, and p < 0.05 was conspicuous.

### DNA Methylation Difference Analysis and DNA Methylation Regulation mRNA Identification

To further understand the reasons for the change of chemokines, the differences analysis of DNA Methylation was performed by limma package between the para-tumour and tumour tissues. In addition, we analyzed the correlation between the degree of methylation and the expression of this mRNA by Pearson. p < 0.05 and |r| >0.3 were considered significant.

### Patient Samples

A total of 24 Wilms tumour tissues and 24 para-tumour tissues from patients undergoing urological surgery in the children’s Hospital of Chongqing Medical University were collected. This study has got the approval of the Ethics Committee of Children’s Hospital of Chongqing Medical University, and all patients and their parents signed informed consent before joining this study. Immediately, specimens were placed in liquid nitrogen and stored at -80°C for further examination.

### Western Blot

Protein samples were lysed in RIPA buffer (Beyotime) supplemented with 1% proteinase inhibitors (Beyotime). The protein concentration was determined using a bicinchoninic acid (BCA) assay kit (Beyotime). Equal amounts of proteins (10 µg) were separated on an 8% SDS-PAGE (sodium dodecyl sulfate-polyacrylamide gel electrophoresis) gel and transferred onto polyvinylidene fluoride (PVDF; Millipore) membranes. The membranes were then blocked in rapid blocking solution and incubated with the primary antibodies overnight at 4°C. The following antibodies were used: CD4 (1:1,000), CD8 (1:1,000), CD3(1:1,000), CX3CL1(1:1000, GAPDH (1:1,000). Finally, the membranes were incubated with HRP−conjugated secondary antibody (1:1,000) for 2 h at room temperature. The immunoblots were visualized using the Immobilon Western Chemiluminescent HRP Substrate, and the bands were quantified, relative to GAPDH, by densitometric analysis (GeneGnome, Syngene UK).

### Immunofluorescence

We co-stained CX3CL1 with T cell markers CD3, CD4 and CD8. The details are as follows. tumour and para-tumour samples were fixed with 4% paraformaldehyde (PFA), dehydrated overnight at 4°C. Paraffin embedding, sectioning, routine deparaffinization, 0.5% BSA blocking and incubated with primary antibody overnight. The primary antibodies were rabbit anti-CD4 (1: 200), rabbit anti-CD8 (1: 200), rabbit anti-CD3 (1: 200), and mouse anti-CX3CL1 (1: 200). CY3 – coupled and FITC - coupled fluorescent secondary antibodies (1:200) were incubated at 25°C for 30 min, and the nucleus was stained with DAPI for 15 min. At least three optical fields were selected for each section for morphological evaluation.

### Statistical Method

Difference analysis was undergoing by edgeR package and limma package. The KM method was performed to evaluate the expression of patients’ chemokines and immune cells and OS. A log-rank test was used to compare the survival differences between the groups. Lasso and multi-factor Cox regression were used to constructing the model, and the ROC curve and correction curve was used for evaluation. The difference of immune cells between tumour tissues and para-tumour was compared by Kruskal.test. Pearson correlation analysis was used. The above statistics were performed using R software (version 4.0.1). Bilateral P < 0.05 was considered statistically significant.

## Results

### Chemokines Are Abnormally Expressed in Wilms Tumour

**Figure 1:**
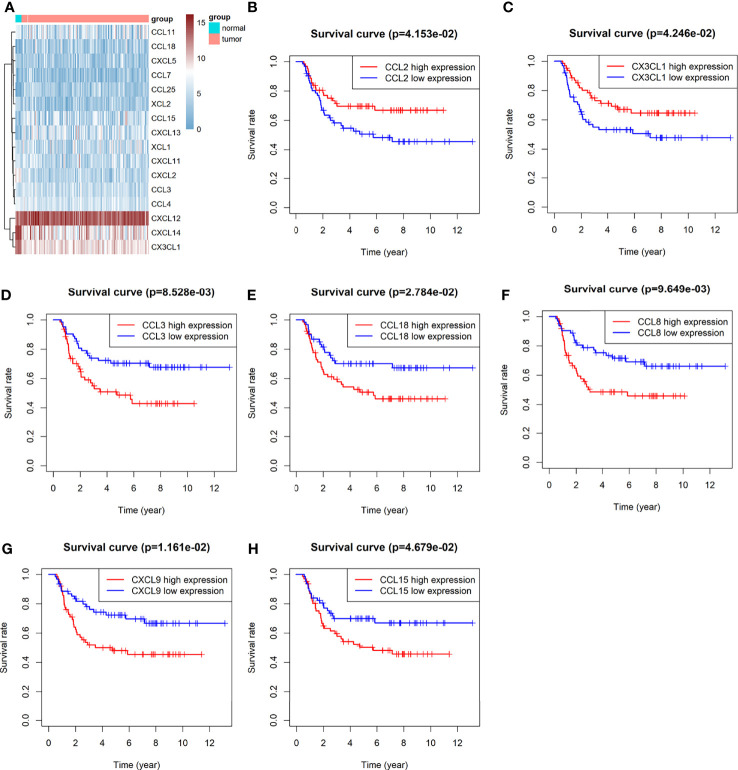
Difference analysis and Kaplan-Meier analysis of Chemokines: **(A)** heat map of significantly differentially expressed chemokines. **(B–H)** KM analysis of chemokines CCL2, CX3CL1,CCL3, CCL8, CCL15, CCL1 z8 and CXCL9.

Of all 32 chemokines, 16 were differentially expressed between Wilms tumour and para-tumour sample (**Figure 1A**) Three chemokines were down-regulated in Wilms tumour samples, and 13 chemokines were up-regulated. Comprehensively evaluating the expression abundance and stability of chemokines, it was found that the three down-regulated chemokines CX3CL1, CXCL14 and CXCL2 had extremely significant differences and high expression abundance.

### Chemokines Are Associated With Prognosis

A total of 7 chemokines were associated with survival, of which CCL2 and CX3CL1 were positively correlated with prognosis, while high expression of CCL3, CCL8, CCL15, CCL18 and CXCL9 predicted poor prognosis (**Figures 1B–H**).

### Establishment of an Chemokines-Based Prognosis Model

**Figure 2:**
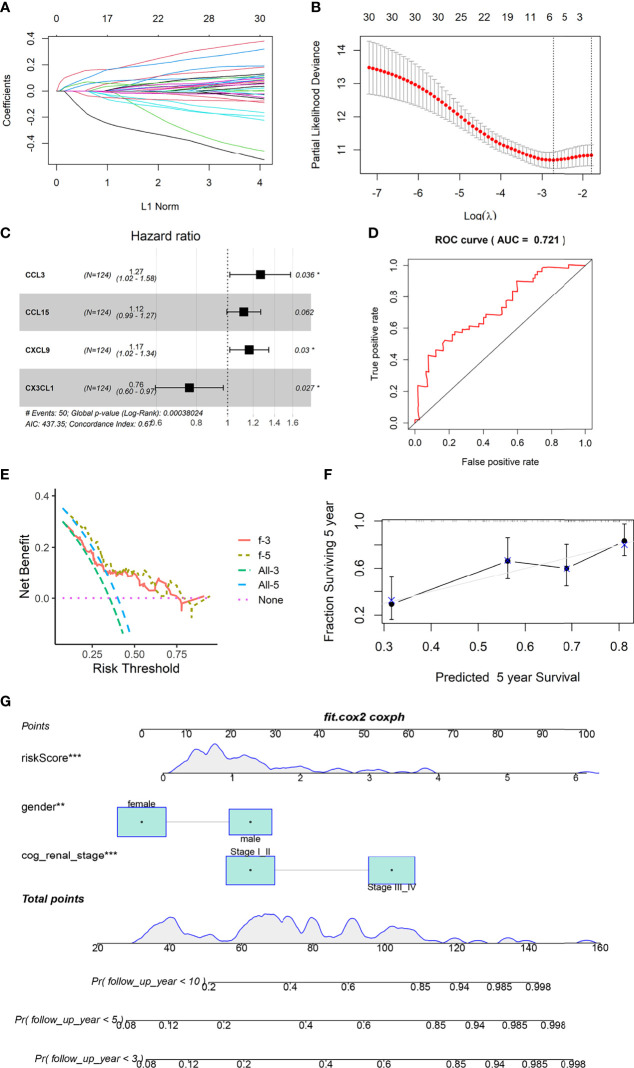
Construction and validation of prognostic model. **(A, B)** LASSO Cox regression analysis based on OS. **(C)** Forest plots presenting the multivariate Cox proportional hazards regression analysis of prognostic chemokines in OS. **(D)** Time-dependent ROC curves. **(E)** DCA curves based on 4 Chemokines of 3 and 5 years. **(F)** Calibration curves for predicting the fitness of the model in 5 years. **(G)** Nomogram shows that the risk scores of the model is independent of clinical data. ** represents P value less than 0.01, *** represents P < 0.001.

Four genes were included in the final model, namely CCL3, CCL15, CXCL9 and CX3CL1 (**Figures 2A–C**). CX3CL1 was associated with a good prognosis, while CCL3, CCL15 and CXCL9 predicted poor prognosis. This is consistent with the previous KM analysis results.

### Verification of the Predictive Power of the Model

The area under the ROC curve was 0.721 (**Figure 2D**). The Calibration curves and DCA curves showed that the prognostic model composed of four chemokines had a good predictive ability (**Figures 2E, F**). Nomograms shows that age and clinical stage are associated with prognosis, and the role of risk scores in judging prognosis is independent of clinical information (**Figure 2G**).

### Low Infiltration of T Cells in Tumors

**Figure 3:**
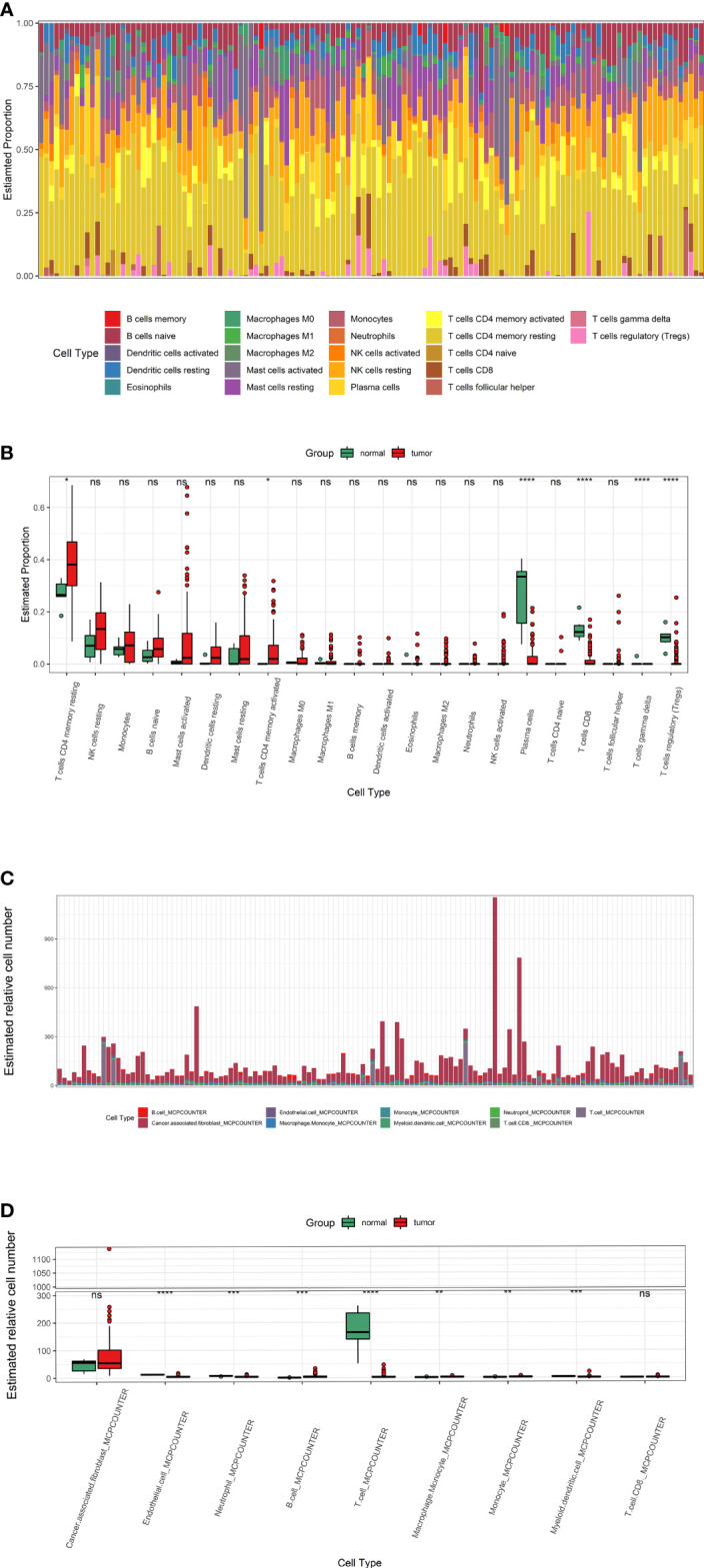
The infiltration of immune cells in Wilms tumors and para-tumors. **(A, B)** The infiltration of immune cells in each sample and Immune cells difference between tumors and para-tumors evaluate by Cibersort algorithm. **(C, D)** The infiltration of immune cells evaluate by MCPcounter. * represents P < 0.05, ** represents P value less than 0.01, *** represents P < 0.001, **** represents P < 0.0001, ns represents P > 0.05.

Cibersort and MCPcounter algorithm calculated the infiltration of 22 and 9 immune cells in Wilms tumour (**Figures 3A, C**). Cibersort algorithm showed that T cells, including CD8+ T cells, CD4+ T cells and regulatory T cells, were lower in tumour tissues than para-tumor tissues. (**Figure 3B**). MCPcounter showed significant different expression of total T cells while CD8+ T cells did not differ between tumour and para-tumour (**Figure 3D**).

### The Number of T Cells Correlates With CX3CL1 Expression

**Figure 4:**
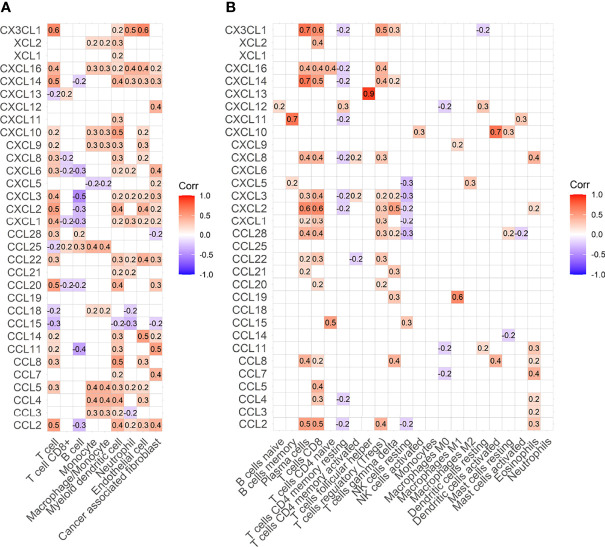
Heat map of correlation between immune cells and chemokines. **(A)** Heat map of correlation between immune cells and chemokines estimated by MCPcounter. **(B)** Heat map of correlation between immune cells and chemokines estimated by Cibersort algorithm.

Most chemokines are positively correlated with immune cells (**Figures 4A, B**). MCPcounter algorithms consistently showed that CX3CL1 was positively correlated with T cells, with a correlation coefficient of 0.6 (**Figure 4A**). Cibersort algorithms showed that CX3CL1 was positively correlated with CD8+ T cells, with a correlation coefficient of 0.6 (**Figure 4B**).

### The Expression of Chemokines Is Regulated by DNA Methylation

**Figure 5:**
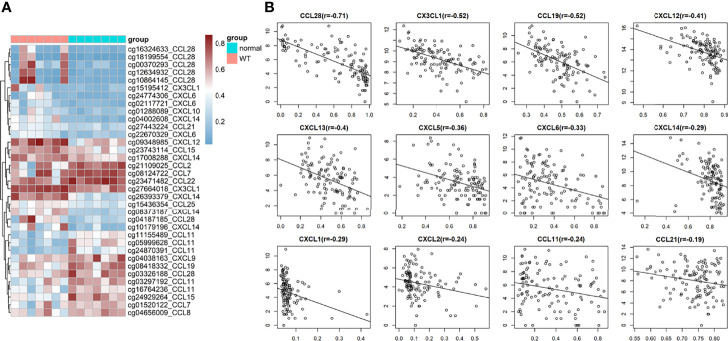
DNA methylation in wilms tumor. **(A)** Heat map of significantly Differential methylation chemokines. **(B)** Correlation between gene expression and DNA methylation.

We found that the DNA methylation state of Wilms tumour tissue was significantly different from that of the para-tumour tissues. Further analysis of the methylation status of chemokines showed that 35 methylation sites were differentially methylated in 19 chemokines (**Figure 5A**). The mRNA expression levels of 12 chemokines correlated with the degree of DNA methylation. It is worth noting that all chemokine methylation levels were negatively associated with mRNA expression (**Figure 5B**). The degree of DNA methylation of CCL28, CX3CL1 and CCL5 was strongly correlated with mRNA expression, with a correlation coefficient of -0.71, -0.52, and-0.52, respectively.

### The Differential Expression and Correlation of CX3CL1 and T Cells Were Verified by WB and Immunofluorescence

**Figure 6:**
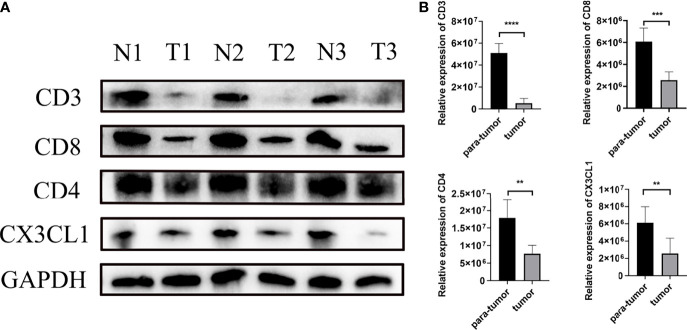
The differential expression of CX3CL1 and CD3, CD4, CD8+ T cells in tumor and para-tumor tissues. **(A)** The WB image of CX3CL1 and CD3, CD4, CD8+ T cells in tumor and para-tumor tissues. **(B)** The summary graph of the expression level of CX3CL1 and CD3, CD4, CD8+ T cells in tumor and para-tumor tissues. ** represents P value less than 0.01, *** represents P < 0.001, **** represents P < 0.0001.

**Figure 7:**
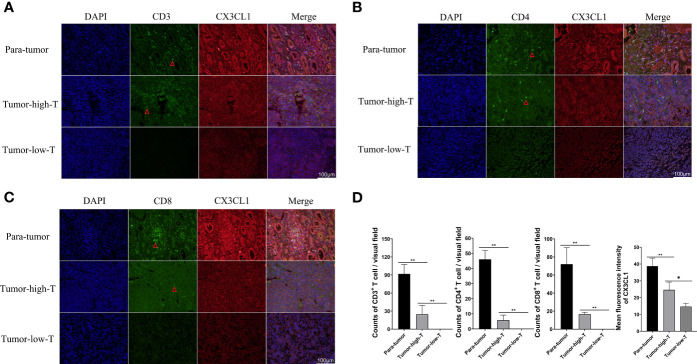
Immunofluorescence of CX3CL1, CD3, CD8 and CD4 positive T cells in high- T cells and low-T cells infiltration tumor tissues and para-tumor tissues. **(A)** The Immunofluorescence of CX3CL1 and CD3+ T cells. **(B)** The Immunofluorescence of CX3CL1 and CD4+ T cells. **(C)** The Immunofluorescence of CX3CL1 and CD8+ T cells. **(D)** The summary graph of the expression level of CX3CL1 and CD3, CD4, CD8+ T cells in tumor and para-tumor tissues [**(A–C)** the scale bar:100μm]. * represents P < 0.05, ** represents P value less than 0.01.

The result of Weston blot showed that CX3CL1 was more abundant in para-tumor samples (**Figure 6**). CD3+ T cells, CD4+ T cells, and CD8+ T cells were lower in tumor samples than para-tumor samples (**Figure 6**). Immunofluorescence also showed that CX3CL1 was highly expressed in para-tumour, mainly around renal tubules (**Figures 7A–C**). This is consistent with previous reports (19, 20). The degree of T cell infiltration was lower in tumor. Most tumor samples were difficult to find T cells, but there were still a small number of tumor samples with high T cells infiltration. These T cells were mainly distributed in the tumor stroma, especially around the blood vessels. In the area with abundant blood vessels, the number of T cells was even higher than the average amount of T cells in kidney (**Figures 7A–C**). The expression of CD4+ and CD8+ subsets was similar to that of total T cells. Additionally, to verify the relationship between T cell infiltration and CX3CL1 expression, we divided tumor tissues into high T cell infiltration and low T cell infiltration. In tumor tissues, the higher the expression of CX3CL1, the higher the number of T cells, whether it is CD8+ T cells or CD4+ T cells (**Figure 7D**).

## Discussion

As a common solid tumour in children, nephroblastoma has made significant progress in treatment. At present, the treatment of nephroblastoma is mainly surgery, radiotherapy and chemotherapy (2). However, radiotherapy and chemotherapy may have serious side effects on children, the long-term impact on patients is unclear (2). Some patients are poorly treated, and new treatments need to be explored. Immunotherapy has shown significant advantages in clinical practice. Drugs based on PD1 and PDL1 block have been successful in many tumours, such as lung cancer and melanoma (21, 22). But there are few reports of childhood tumours. Previous studies have shown that a significant increase in plasma EV PD-L1 in WT patients contributes to the immunosuppression of peripheral CD8+ T cells (23). This suggests that nephroblastoma also has immunosuppression similar to that in adult tumors. However, children with Wilms tumors are faced with another major problem, low T cell infiltration. Therefore, the effect of immune checkpoint inhibitors alone may not be ideal. Although we found that a small number of patients also have rich immune cell infiltration, accurate selection of such patients for individual immune checkpoint inhibition therapy may have some effect. But for most nephroblastoma patients, increasing immune cells infiltration in tumor is the key to immunosuppressive therapy. After all, if there is no T cells, how can the function of T cells be enhanced by immune checkpoint inhibitors? in fact, Studies have shown that the therapeutic effect of immunosuppressants is related to tumour immune checkpoints and the infiltration degree of immune cells (24).

To solve the problem of low infiltration of immune cells in tumour. We focused our attention on chemokines. Chemokines are named for their chemotaxis to immune cells. Numerous studies have shown that chemokines regulate immune cell trafficking in tumours and are associated with tumour development, progression and angiogenesis (16, 25). Chemokines can recruit tumour-associated macrophages and regulatory T cells to promote tumour progression, which is related to poor prognosis (26–28). At the same time, chemokines can also recruit CD8+ T cells to inhibit tumour progression (29, 30). For example, chemokines can increase the aggregation of CD8+ T cells in melanoma, thereby promoting immune monitoring and controlling cancer growth (30). CX3CL1 exerts antitumor activity through NK cells and T cells (16). CCL7 is a key chemokine that recruits dendritic cells (DCs) (25). This role of chemokines undoubtedly laid the foundation for immunotherapy.

In this study, we performed a comprehensive analysis of chemokines in nephroblastoma. We found that nearly half of the chemokines were abnormally expressed in nephroblastoma. The expression of CX3CL1, CXCL14 and CXCL2 in nephroblastoma was significantly decreased. Further, we explored the chemokines related to prognosis. CCL2 and CX3CL1 were positively correlated with prognosis, while the high expression of CCL3, CCL8, CCL15, CCL18 and CXCL9 predicted a poor prognosis.

To explore the immune infiltration of nephroblastoma, we evaluated the immune cells both by MCPcounter and Cibersort algorithm. We found that the expression of total T cells in tumours was significantly reduced. Further analysis of the T-cell subpopulation found that the CD8+ T cell infiltration calculated by Cibersort was significantly reduced in tumours, while MCPcounter showed no difference. To identify whether T cells differ in the Wilms tumour and para-tumour, We experimentally verified by WB and immunofluorescence. The expression of CD3 in the tumour was significantly lower than that in para-tumour, indicating that total T cells were poorly infiltrated in tumours. Furthermore, we performed WB and immunofluorescence to investigate the expression and distribution of CD3+, CD4+ and CD8+ T cells in the tumour and para-tumour tissues. It was found that CD8+ T cells were scattered around renal, but almost no distribution in most tumour tissues. This is a little different from the previous calculation of MCPcounter which showed that CD8+ T cells was no differentially expression in the tumour and para-tumour tissues,while identical to the Cibersort algorithm. These results suggest that, In Wilms tumour,Cibersort can be used to assess immune cells Infiltration more accurately than MCPcounter. There are still a small number of patients with abundant immune cell infiltration, and these immune cells are mainly distributed in tumor stroma, especially in areas rich in blood vessels.

To explore the chemotaxis of chemokines to immune cells, we conducted a correlation analysis between chemokines and immune cells. Whether MCPcounter or Cibersort algorithm, It was found that CX3CL1 was strongly correlated with total T cells. Cibersort algorithm shows that CX3CL1 was positively correlated with CD8+ T cells with a correlation coefficient of 0.6. In addition, we found that T cells were positively correlated not only with CX3CL1 but also with CCL2, CXCL12 and other chemokines, indicating that a variety of chemokines can show chemotaxis to T cells. Co-staining confirmed that CX3CL1 strongly expressed tumor tissues had more T cell infiltration.

The expression of chemokines is affected by many pathways; NF-kappa B, NOTCH, MYC or WNT are related to the expression of chemokines (27, 31). It has been reported that TNFα, IL-1β, and LPS can stimulate the expression of CX3CL1 (32). The hypoxia microenvironment of tumours affects the expression of chemokines (33). In addition, DNA methylation plays an essential role in the expression of chemokines (34). It has been reported that DNA methylation is the main negative regulatory mechanism of CCL5, and low-dose DNA methyltransferase inhibitor 5 - azacytidine can affect the expression of chemokines and immune infiltration (35). Here, we found that CX3CL1 was down-regulated in Wilms’ tumour. To explore the reasons for the abnormal expression of chemokines, we analyzed the methylation of gene expression sites of chemokines. It was found that the methylation of chemokine DNA was significantly different in tumours and para-tumour tissue, and the CX3CL1 gene was seriously hypermethylated at cg27664018 in Wilms tumour tissues. Further correlation analysis between methylation level and mRNA expression was performed. The degree of methylation of all chemokines was negatively correlated with mRNA, which was consistent with the negative regulation of gene expression by DNA methylation (36). In particular, the correlation between the methylation BETA value of CX3CL1 and the expression of CX3CL1 reached -0.52. This evidence provides a basis for applying DNA methylation inhibitors in tumour immunotherapy.

In summary, this study found a deficiency of T cells and other immune cells in Wilms tumours. This phenomenon may be caused by the lack of chemokines such as CX3CL1. Furthermore, we found that chemokine deficiency was associated with DNA hypermethylation. Regulating immune cell infiltration by regulating chemokine expression with DNA methylation inhibitors may provide a new approach for treating nephroblastoma in the future.

## Data Availability Statement

The datasets presented in this study can be found in online repositories. The names of the repository/repositories and accession number(s) can be found in the article/**Supplementary Material**.

## Ethics Statement

The studies involving human participants were reviewed and approved by The Institutional Review Board, Children’s Hospital of Chongqing Medical University. Written informed consent to participate in this study was provided by the participants’ legal guardian/next of kin. Written informed consent was obtained from the individual(s), and minor(s)’ legal guardian/next of kin, for the publication of any potentially identifiable images or data included in this article.

## Author Contributions

Conceptualization: (TM, LJ, DH); Data curation: (TM, LJ, ZZ, DH); Formal analysis: (LJ, JW, ML, CZ); Funding acquisition: (DH); Methodology: (XJT, ZW, XMT, BX); Writing—original draft: (TM, ZZ, DH); Resources: (DH); Supervision: (DH). All authors contributed to the article and approved the submitted version.

## Funding

The present study was supported by the Special Project of Science and Technology Innovation for Social Undertakings and Livelihood Guarantee of Chongqing (Grant Nos. cstc2019jscx-tjsbX0003 and cstc2017shmsA130103).

## Conflict of Interest

The authors declare that the research was conducted in the absence of any commercial or financial relationships that could be construed as a potential conflict of interest.

## Publisher’s Note

All claims expressed in this article are solely those of the authors and do not necessarily represent those of their affiliated organizations, or those of the publisher, the editors and the reviewers. Any product that may be evaluated in this article, or claim that may be made by its manufacturer, is not guaranteed or endorsed by the publisher.
