# Supplementary figures and images for: DNA Hypermethylation-Regulated CX3CL1 Reducing T Cell Infiltration Indicates Poor Prognosis in Wilms Tumour
Source: Front Oncol. 2022 Apr 22;12:882714. doi: 10.3389/fonc.2022.882714 (PMC9072742; doi:10.3389/fonc.2022.882714)

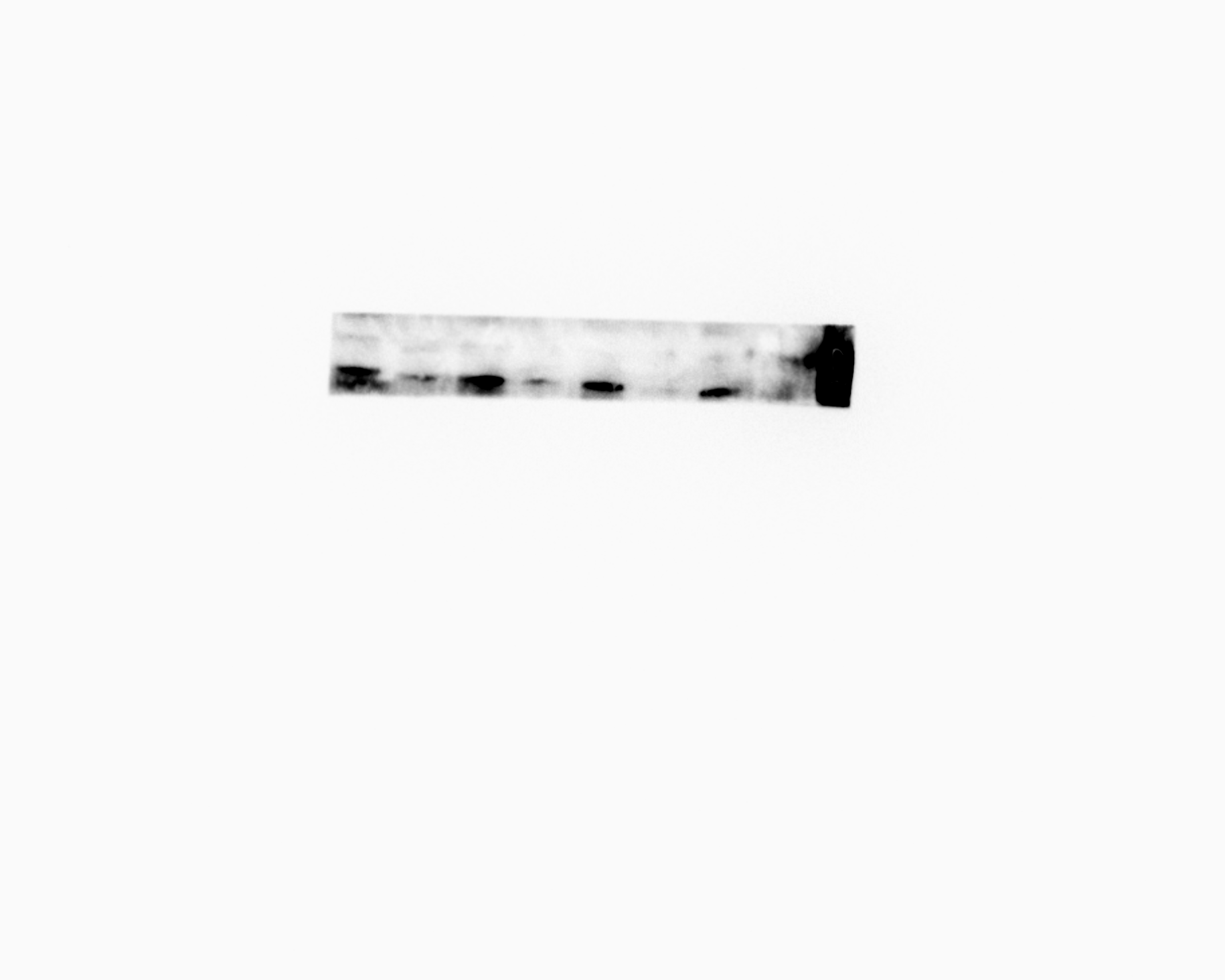

Supplement: Supplementary file 1 [file DataSheet1.zip › WB/1 fig6A CD3.tif]

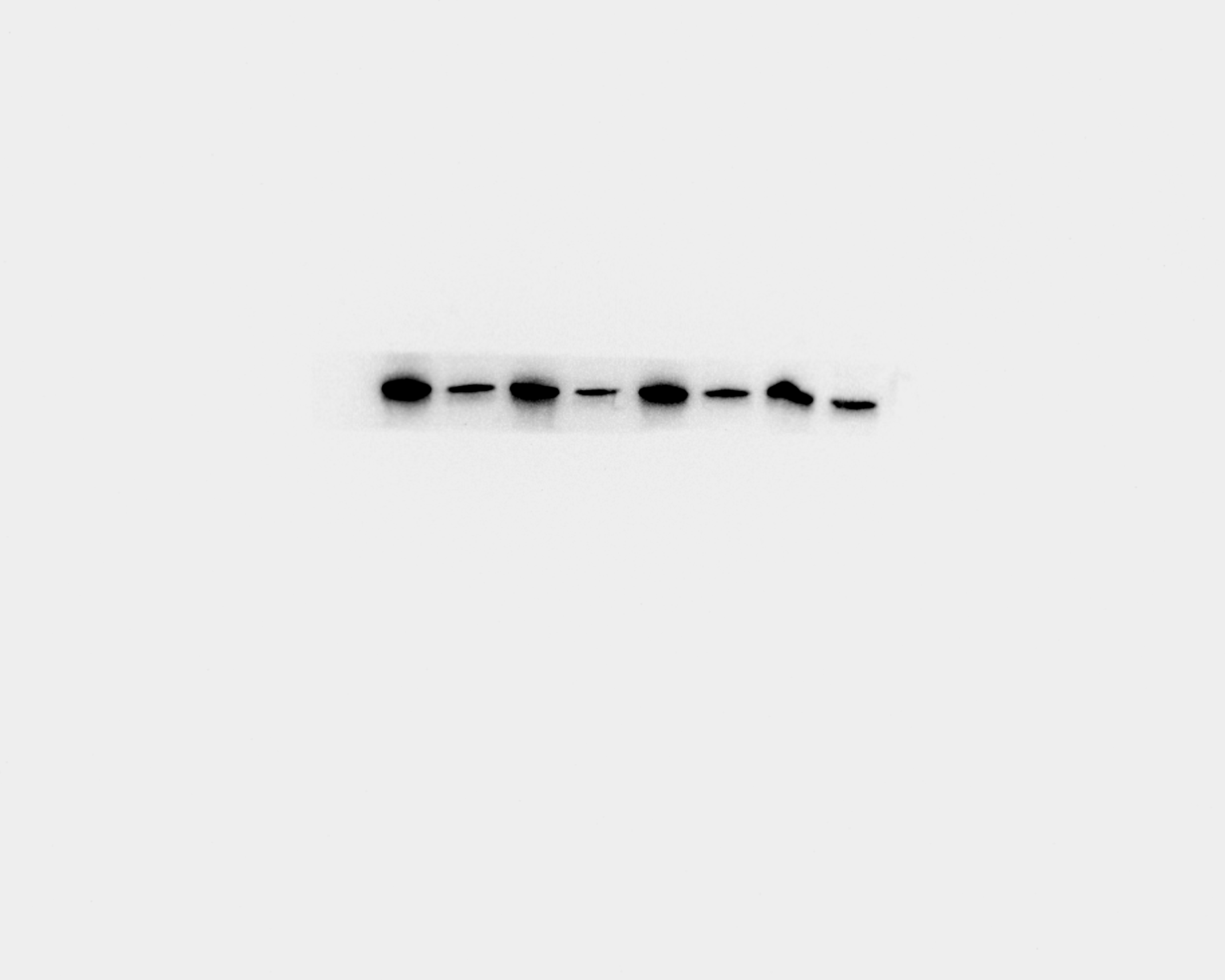

Supplement: Supplementary file 1 [file DataSheet1.zip › WB/2 fig6A CD8.tif]

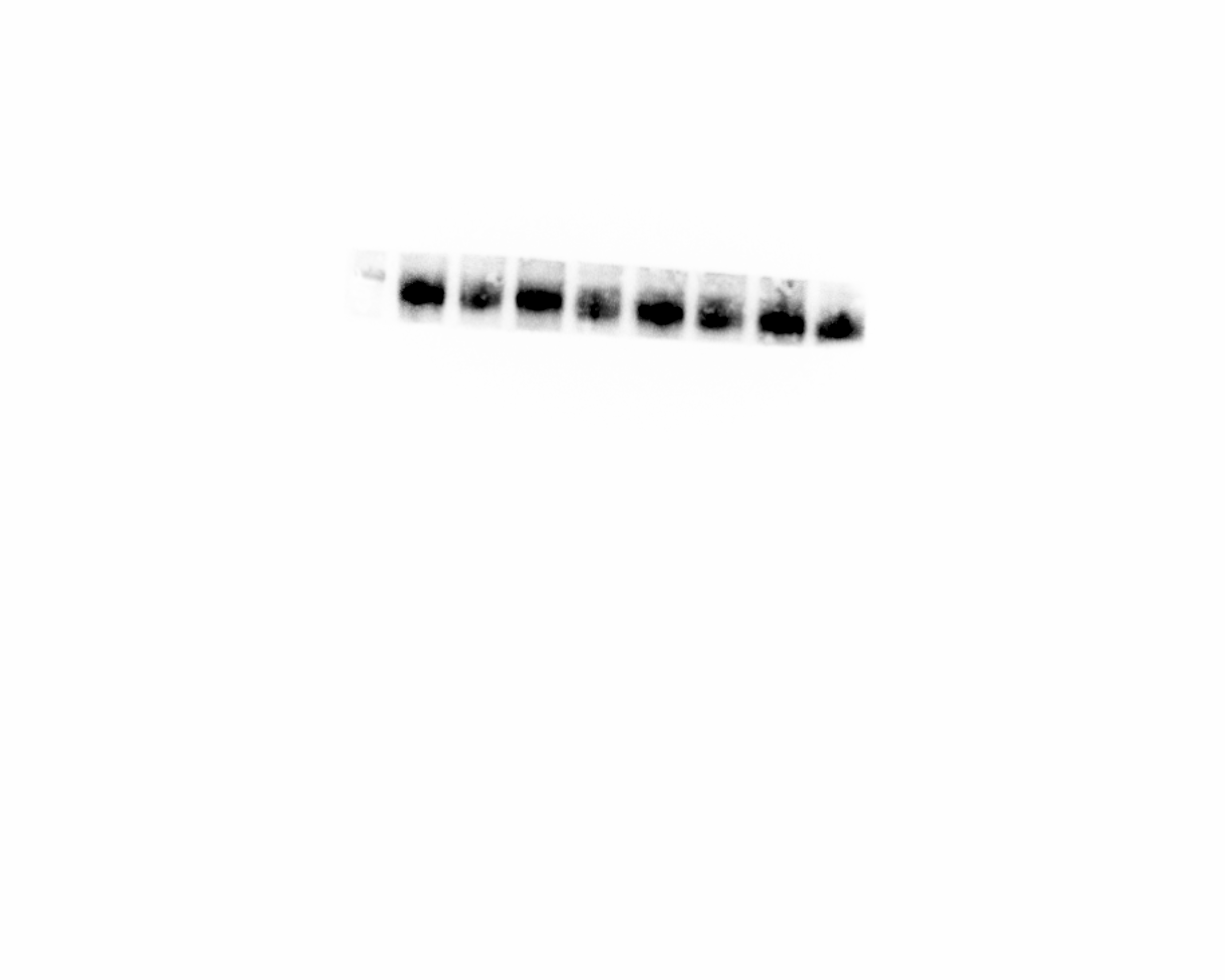

Supplement: Supplementary file 1 [file DataSheet1.zip › WB/3 fig6A CD4.tif]

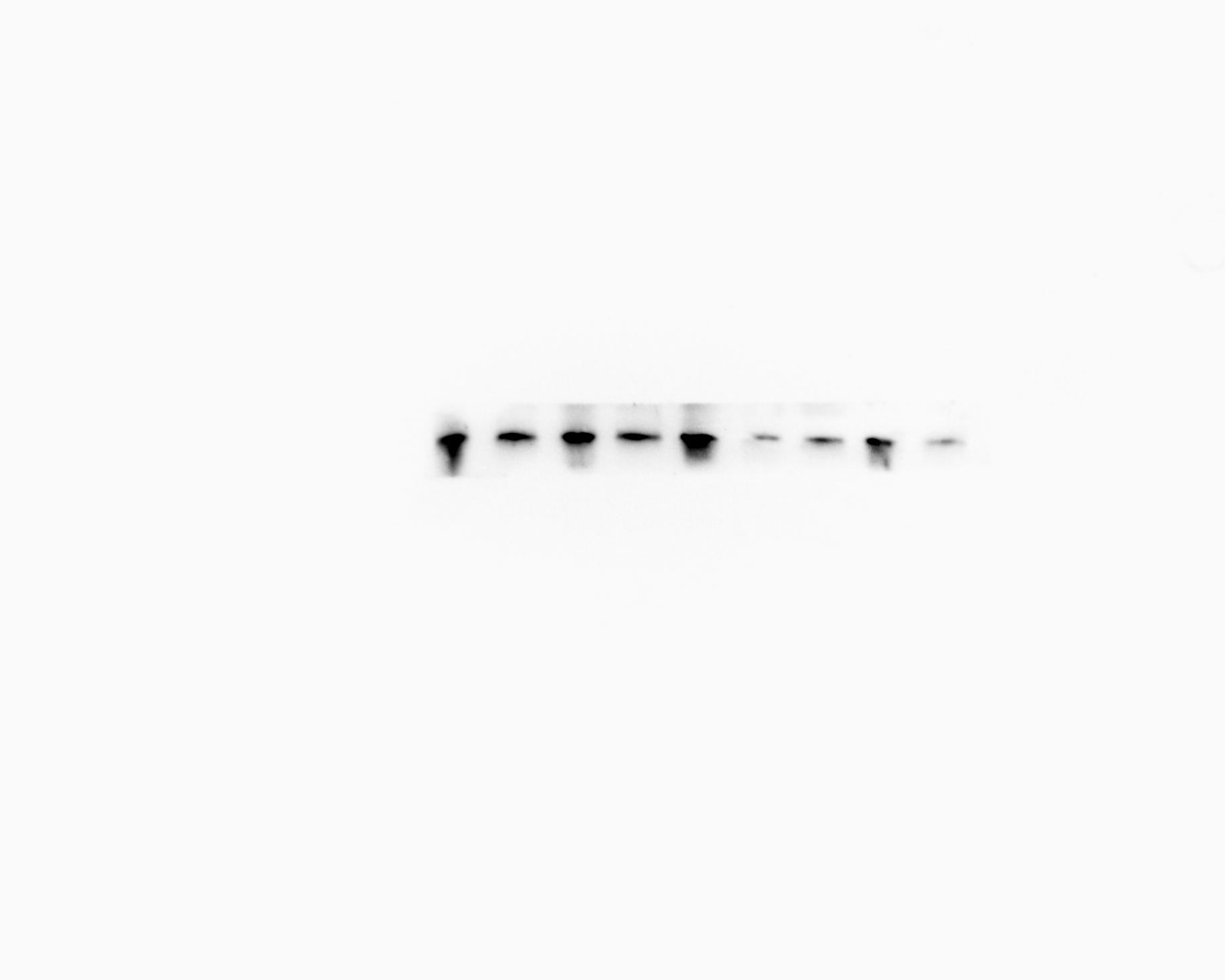

Supplement: Supplementary file 1 [file DataSheet1.zip › WB/4 fig6A CX3CL1.tif]

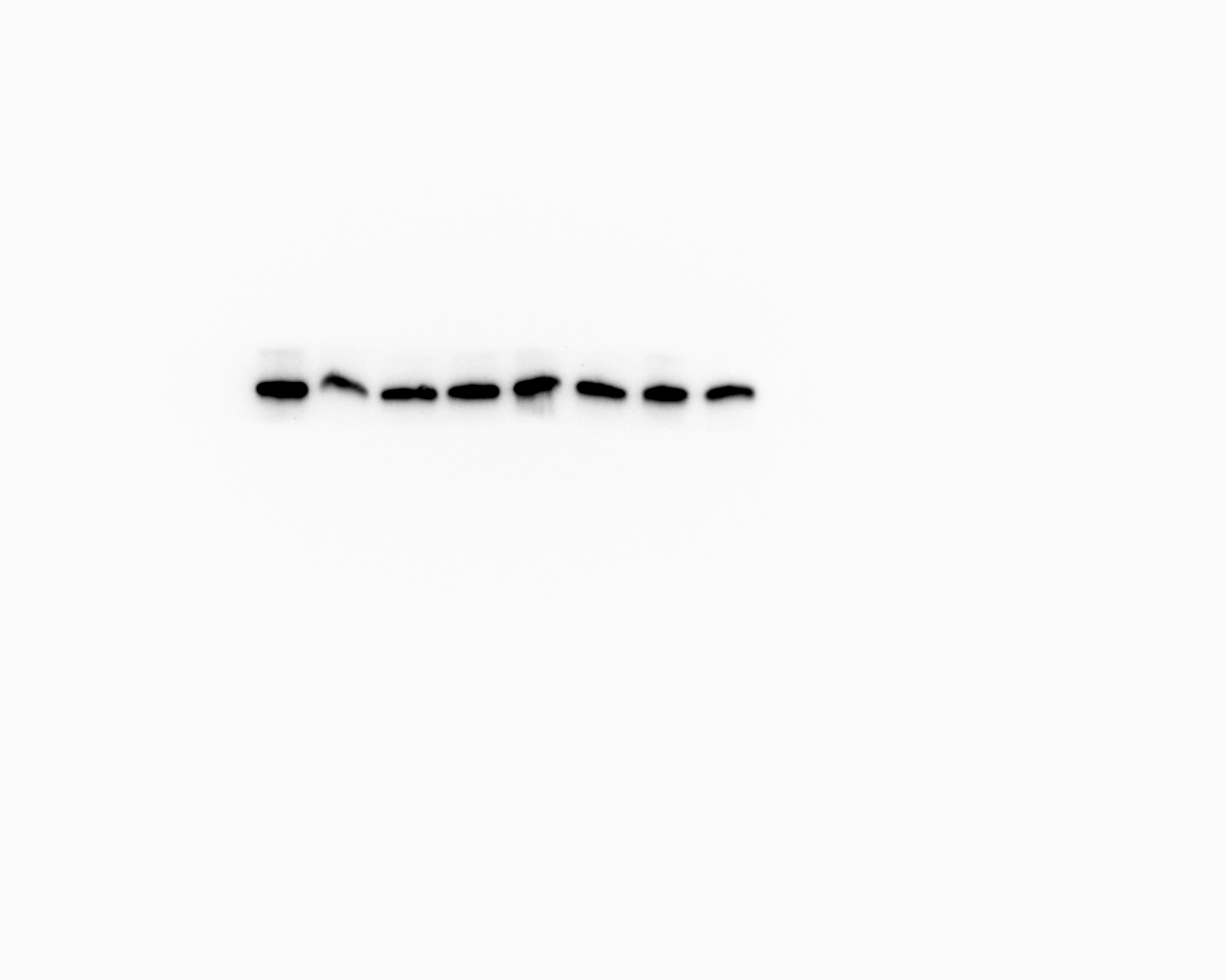

Supplement: Supplementary file 1 [file DataSheet1.zip › WB/fig6A GAPDH.tif]
